# Outbreak of Leptospirosis after Flood, the Philippines, 2009

**DOI:** 10.3201/eid1801.101892

**Published:** 2012-01

**Authors:** Al-shere T. Amilasan, Mugen Ujiie, Motoi Suzuki, Eumelia Salva, Maria Cecilia P. Belo, Nobuo Koizumi, Kumiko Yoshimatsu, Wolf-Peter Schmidt, Shane Marte, Efren M. Dimaano, Jose Benito Villarama, Koya Ariyoshi

**Affiliations:** San Lazaro Hospital, Manila, Republic of the Philippines (A.T. Amilasan, E. Salva, M.C.P. Belo, S. Marte, E.M. Dimaano, J.B. Villarama);; National Center for Global Health and Medicine, Tokyo, Japan (M. Ujiie);; Nagasaki University, Nagasaki, Japan (M. Ujiie, M. Suzuki, W.-P. Schmidt, K. Ariyoshi);; National Institute of Infectious Diseases, Tokyo (N. Koizumi);; Hokkaido University, Sapporo, Japan (K. Yoshimatsu)

**Keywords:** flood, leptospirosis, Leptospira, outbreak, the Philippines, Metro Manila, typhoon, Weil disease, bacteria

## Abstract

After a typhoon in September 2009, an outbreak of leptospirosis occurred in Metro Manila, the Philippines; 471 patients were hospitalized and 51 (10.8%) died. A hospital-based investigation found risk factors associated with fatal infection to be older age, hemoptysis, anuria, jaundice, and delayed treatment with antimicrobial drugs.

Leptospirosis is highly endemic to the Philippines. Outbreaks usually occur during the typhoon season (July–October) (*1**–**3*). On September 26, 2009, a typhoon caused serious flooding in Metro Manila (*4*). Starting in the first week of October, the number of patients with suspected signs and symptoms of leptospirosis increased sharply. Until mid-November, 2,299 patients, including 178 who died (case-fatality ratio [CFR] 8%), in 15 hospitals in Metro Manila were reported to the Department of Health (*4**,**5*). For this outbreak, we conducted a hospital-based investigation to describe the characteristics of hospitalized patients, investigate risk factors for death, and identify the causative *Leptospira* species and serogroups.

## The Study

We conducted our investigation at San Lazaro Hospital, a national 500-bed referral infectious disease center for Metro Manila and neighboring provinces, which mostly serves economically disadvantaged persons. On October 11, 2009, this hospital initiated prospective surveillance and retrospective data collection. The study ended on October 31, when cases had decreased to baseline level (<1 case/d). The list of patients was obtained from the inpatient database with International Classification of Diseases, 10th revision, coding. Vital sign information and laboratory findings at admission were collected from medical charts.

Eligible were all hospitalized patients who had 1) fever plus at least 2 other signs and symptoms of leptospirosis (headache, myalgia, eye pain, nausea, vomiting, abdominal pain, diarrhea, conjunctival suffusion, jaundice, tea-colored urine, oliguria, anuria, or unusual bleeding) (*6*); 2) history of wading in floodwater; and 3) symptoms that started September 26–October 31. We evaluated prognostic factors and the effect of therapeutic factors by using univariate and multivariate Poisson regression analyses with robust SEs (*7*).

A total of 486 cases met clinical criteria for leptospirosis; 15 were excluded because of insufficient data. Patients were predominantly young and male (Table 1). The most common clinical features were conjunctival suffusion and myalgia, followed by abdominal pain and oliguria. Among 471 patients, 51 died (CFR 10.8%); 12 (2.6%) were discharged before improvement; and 7 (1.5%) were transferred to other hospitals, mainly for dialysis. Primary causes of death were pulmonary hemorrhage (18 [35%]) and acute respiratory distress syndrome/severe respiratory failure (12 [24%]), followed by acute renal failure (10 [20%]) and multiple organ failure/disseminated intravascular coagulation (8 [16%]). Mean time ± SD from illness onset to death was 7.7 ± 5 days; 36 (71%) died within 2 days of admission.

**Table 1:** Characteristics of 471 leptospirosis patients, San Lazaro Hospital, Manila, the Philippines, 2009*

Characteristic	No. (%) patients total, n = 471	No. (%) patients who died, n = 51	Univariate analysis	
			Risk ratio	95% CI
Sex				
M	424 (90)	45 (88.2)	Reference	
F	47 (10)	6 (11.8)	1.2	0.54–2.67
Age >30 y†	235 (49.9)	37 (72.6)	2.65	1.47–4.78
Low BMI, <20, n = 398	129 (32.4)	16 (35.6)	1.15	0.65– 2.04
Clinical features, n = 471				
Headache	246 (52.2)	19 (37.3)	0.54	0.32–0.93
Myalgia	361 (76.7)	36 (70.6)	0.73	0.42–1.29
Cough	83 (17.6)	3 (5.6)	0.29	0.09–0.92
Malaise	208 (44.2)	23 (45.1)	1.04	0.62–1.75
Vomiting	272 (57.8)	22 (43.1)	0.56	0.33–0.94
Abdominal pain	288 (61.2)	28 (54.9)	0.77	0.46–1.3
Diarrhea	192 (40.8)	24 (47.1)	1.29	0.77–2.17
Conjunctival suffusion	368 (78.1)	41 (80.4)	1.15	0.6–2.21
Jaundice	225 (47.8)	38 (74.5)	3.2	1.74–5.84
Tea-colored urine	156 (33.1)	17 (33.3)	1.01	0.58–1.75
Oliguria	286 (60.7)	27 (52.9)	0.73	0.43–1.22
Anuria	26 (5.5)	10 (19.6)	4.17	2.37–7.36
Hemoptysis	15 (3.2)	7 (13.7)	4.84	2.63–8.9
Skin hemorrhage	2 (0.4)	0	NA	
Convulsion	3 (0.6)	1 (2)	3.12	0.62–15.82
Dizziness	43 (9.1)	1 (2)	0.2	0.03–1.41
Vital status at admission				
Hypotension, <100 mm Hg, n = 455	177 (38.9)	14 (28.6)	0.63	0.35–1.13
Tachycardia, >100 beats/min, n = 461	84 (18.2)	14 (27.5)	1.7	0.96–3.0
Tachypnea, >20 breaths/min, n = 460	253 (55)	34 (66.7)	1.64	0.94–2.84
Initial laboratory findings				
Neutrophilia, >12 x 10^9^ cells/L, n = 430	171 (39.8)	18 (58.1)	2.1	1.05–4.17
Thrombocytopenia, <50 x 10^3^ cells/L, n = 426	50 (11.7)	10 (33.3)	3.76	1.87–7.57
AST >100 IU/L, n = 220	44 (20)	2 (28.6)	1.6	0.32–8.0
ALT >100 IU/L, n = 220	34 (15.5)	2 (28.6)	2.2	0.44–10.9
BUN >80 mg/dL, n = 385	185 (48.1)	12 (75)	3.24	1.06–9.89
Cr >3.0 mg/dL, n = 413	183 (44.3)	13 (72.2)	3.27	1.19–9.01

*BMI, body mass index; NA, not applicable; AST, aspartate aminotransferase; ALT, alanine transaminase; BUN, blood urea nitrogen; Cr, creatinine. †Mean ± SD 31.3 ± 13.1 for total patients, 39.5 ± 13.6 for patients who died; risk ratio (95% CI) 1.04 (1.02–1.06).

Univariate analysis showed the following to be associated with death: older age, jaundice, anuria, and hemoptysis (Table 1). Of the initial laboratory findings, neutrophilia, thrombocytopenia, increased blood urea nitrogen, and increased creatinine levels were associated with death. Most patients received antimicrobial drugs (mainly penicillin G) as first-line treatment. Delayed initiation of treatment increased risk for death (Table 2). No patient received prophylactic antimicrobial therapy. Administration of rapid volume replacement therapy and diuretics was associated with death but probably reflected severe renal disease. Only 1 patient received peritoneal dialysis and 1 received mechanical ventilation.

**Table 2:** Effects of therapeutic approaches on death from leptospirosis, San Lazaro Hospital, Manila, the Philippines, 2009

Therapeutic factor	Total cases, n = 471*	Fatal cases, n = 51*	Univariate analysis			Multivariate analysis	
			Risk ratio	95% CI		Risk ratio	95% CI
Days from onset to first antimicrobial drug therapy, n = 466	4.9 ± 2.6	5.6 ± 2.8	1.09	1.01–1.18		1.09†	1.00–1.17
<7	351 (75.3)	31 (63.3)	Reference			Reference	
>7	115 (24.7)	18 (36.7)	1.77	1.03–3.05		1.76†	1.03–3.01
First antimicrobial agent used, n = 469							
Penicillin G	434 (92.5)	47 (94)	Reference			Reference	
Ceftriaxone	10 (2.1)	2 (4)	0.79	0.26–2.42		0.62‡	0.16–2.38
Doxycycline	9 (1.9)	0 (0)					
Others	16 (3.4)	1 (2)					
Days from onset to admission†	5.1 ± 2.6	5.7 ± 3.2	1.08	1.0–1.16		1.08†	1.0–1.16
Rapid volume replacement therapy							
Performed	334 (70.9)	46 (90.2)	3.77	1.53–9.3		2.63§	0.6–11.4
Not performed	137 (29.1)	5 (9.8)	Reference			Reference	
Diuretics							
Used	356 (75.6)	46 (90.2)	2.97	1.21–7.31		1.15§	0.31–4.23
Not used	115 (24.4)	5 (9.8)	Reference			Reference	

*Values are no. (%) or mean ± SD. †Adjusted for age group (<30 or >30 y). ‡Adjusted for age group and duration from symptom onset to antimicrobial therapy initiation. §Adjusted for age group and creatinine level.

During the outbreak, 2 kinds of rapid diagnostic tests for leptospirosis—Leptospira Serology Kit (Bio-Rad, Marnes-la-Coquette, France) and PanBio IgM ELISA (Panbio Diagnostics, Brisbane, Queensland, Australia)—were available in the hospital, although the number of kits was limited. Plasma collection for additional laboratory confirmation started October 11. Samples were initially stored at –4°C in the hospital laboratory and then frozen at –40°C in the National Reference Laboratory, STD/AIDS Cooperative Central Laboratory, San Lazaro Hospital. In June 2010, microscopic agglutination test (MAT) and PCR were performed in the National Institute of Infectious Diseases, Tokyo, Japan, as described (*8**,**9*). A case was defined as laboratory confirmed if 1) specific antibodies were detected with titer >400 or at least a 4-fold increase in reciprocal MAT titer between paired samples, 2) PCR for *Leptospira flaB* gene was positive for at least 1 blood sample, or 3) rapid diagnostic test result (either test) was positive for at least 1 blood sample.

A total of 134 plasma samples were collected from 93 patients. From 25 patients, 2–4 samples were collected; mean time from first to last collection was 6.1 (range 1–18) days. Among the 93 patients, 15 had a single MAT titer >400 and 4 showed a 4-fold increase in the MAT titer in paired samples. Most antibodies were against *L. borgpetersenii* serovar Tarassovi, followed by serovars Poi and Sejroe and *L. interrogans* serovars Losbanos and Manilae. PCR produced positive results for 4 patients, 2 of whom had a negative MAT titer. PCR-detected *Leptospira* strains were phylogenetically characterized by the *flaB* sequence (*8**,**9*); 3 were identical to those of isolates from rats in Metro Manila, but 1 was distinct (Figure).

**Figure:**
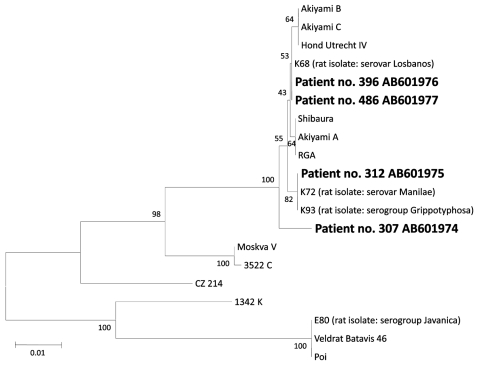
Phylogenetic tree based on the *Leptospira flaB* gene sequence. The sequences obtained in this study are indicated in **boldface** and have been deposited in DDBJ/GenBank/EMBL (accession numbers indicated). The sequences of rat isolates are derived from a previous study (*9*). Sequence alignments were conducted by using MEGA4 software and ClustalW (www.megasoftware.net), and phylogenetic distances were calculated by using MEGA4 software and the neighbor-joining method. All 4 PCR products belong to *L. interrogans*; 2 are closely related to serovar Losbanos, 1 to serovar Manilae, and 1 is not associated with strains or serovars included in our analysis. Microscopic agglutination test results indicate infecting serogroups. *L. borgpetersenii* serovar Tarassovi had the highest titer in patients 307 and 486, but this finding can be explained by a cross-reaction. Scale bar indicates nucleotide substitutions per site.

Rapid-test results were available for 85 case-patients; 48 of 78 samples were positive by Leptospira Serology Kit and 5 of 7 were positive by ELISA. Of 27 cases tested by MAT and Leptospira Serology Kit, 6 were positive by both tests. One sample was negative by MAT and ELISA. Taken together, 149 (32%) of all cases underwent any diagnostic testing for leptospirosis, and results for 67 (45%) of these were laboratory confirmed. Clinical and laboratory findings of laboratory-confirmed and suspected cases, respectively, were almost identical except for the presence of jaundice (61.2% vs. 45.5%; p = 0.018), convulsions (4.5% vs. 0%; p = 0.003), and death (3% vs. 12.1%; p = 0.03). No plasma sample test results have been validated; thus, deduced incidence should be considered with caution. To explore the possibility of hantavirus co-infection contributing to the high CFRs, we screened samples for antibodies against hantavirus (*10*), but all were negative.

## Conclusions

Risk factors for fatal leptospirosis were jaundice, anuria, and hemoptysis at admission. These are typical signs of the severe form of leptospirosis called Weil disease (*11*), confirming earlier work (*12**,**13*). Hemoptysis with high CFR (7 [47%] of 15) may have represented leptospirosis-associated severe pulmonary hemorrhagic syndrome (*14*). Some clinical features, including cough, seemed to be associated with lower risk for death, but minor symptoms in dying patients might have been overlooked. High leukocyte counts, blood urea nitrogen and creatinine levels, and lower platelet counts were also associated with death.

Deaths could be reduced if these indicators are detected and appropriate management introduced early. Laboratory data such as potassium level might also be helpful, but these tests are often not available for all suspected cases in resource-poor settings, especially during outbreaks. The trend of most patients being male is compatible with previous reports and thought to reflect higher exposure to contaminated water (*6**,**12**–**14*).

Although all patients received antimicrobial therapy, a considerable proportion died of acute respiratory distress syndrome and acute renal failure within only 2 days of admission, indicating that most patients sought care too late. The World Health Organization recommends starting antimicrobial therapy before the fifth day of disease onset (*6*), but for half of the case-patients reported here, it was started after the fifth day. Our results support the benefits of early initiation of antimicrobial therapy.

Renal failure was clearly a cause of death, but only 1 patient received peritoneal dialysis and another 7 were transferred to other hospitals for hemodialysis after only 3.4 days of hospitalization. This lack or delay of dialysis might have affected outcomes. San Lazaro Hospital is not sufficiently equipped for intensive care. Expanded access to peritoneal dialysis might reduce deaths from severe leptospirosis complicated by acute renal failure.

Leptospirosis is typically seen in resource-poor settings, where costly medical equipment such as dialyzers and ventilators are rarely accessible. For leptospirosis outbreak control and CFR reduction in leptospirosis-endemic regions, continuous case and environmental monitoring and early introduction of appropriate treatment for suspected cases are warranted.

## References

[1] Victoriano AF, Smythe LD, Gloriani-Barzaga N, Cavinta LL, Kasai T, Limpakarnjanarat K, Leptospirosis in the Asia Pacific region. BMC Infect Dis. 2009;9:147. 10.1186/1471-2334-9-147 19732423 PMC2749047

[2] Yanagihara Y, Villanueva SY, Yoshida S, Okamoto Y, Masuzawa T. Current status of leptospirosis in Japan and Philippines. Comp Immunol Microbiol Infect Dis. 2007;30:399–413. 10.1016/j.cimid.2007.05.003 17614131

[3] Pappas G, Papadimitriou P, Siozopoulou V, Christou L, Akritidis N. The globalization of leptospirosis: worldwide incidence trends. Int J Infect Dis. 2008;12:351–7. 10.1016/j.ijid.2007.09.011 18055245

[4] Republic of the Philippines, National Disaster Coordinating Council. Situation report No. 52 on Tropical “Ondoy” (Ketsana) glide no. TC-2009–000205-PHL and Typhoon “Pepeng” (Parma) glide no. TC-2009–000214-PHL [cited 2011 Nov 4]. http://reliefweb.int/sites/reliefweb.int/files/resources/D87F541373D245BC4925767A000C5761-Full_Report.pdf

[5] Asian Disaster Reduction Center. Philippines’ country profile 2009 [cited 2011 Nov 4]. http://www.adrc.asia/countryreport/PHL/2009/PHL2009.pdf

[6] World Health Organization, International Leptospirosis Society. Human leptospirosis: guidance for diagnosis, surveillance and control. Geneva: The Organization; 2003.

[7] Barros AJ, Hirakata VN. Alternatives for logistic regression in cross-sectional studies: an empirical comparison of models that directly estimate the prevalence ratio. BMC Med Res Methodol. 2003;3:21. 10.1186/1471-2288-3-21 14567763 PMC521200

[8] Villanueva SY, Ezoe H, Baterna RA, Yanagihara Y, Muto M, Koizumi N, Serologic and molecular studies of *Leptospira* and leptospirosis among rats in the Philippines. Am J Trop Med Hyg. 2010;82:889–98. 10.4269/ajtmh.2010.09-0711 20439972 PMC2861393

[9] Koizumi N, Muto M, Yamamoto S, Baba Y, Kudo M, Tamae Y, Investigation of reservoir animals of *Leptospira* in the northern part of Miyazaki Prefecture. Jpn J Infect Dis. 2008;61:465–8. 19050356

[10] Truong TT, Yoshimatsu K, Araki K, Lee BH, Nakamura I, Endo R, Molecular epidemiological and serological studies of hantavirus infection in northern Vietnam. J Vet Med Sci. 2009;71:1357–63. 10.1292/jvms.001357 19887743

[11] Bharti AR, Nally JE, Ricaldi JN, Matthias MA, Diaz MM, Lovett MA, Leptospirosis: a zoonotic disease of global importance. Lancet Infect Dis. 2003;3:757–71. 10.1016/S1473-3099(03)00830-2 14652202

[12] Dupont H, Dupont-Perdrizet D, Perie JL, Zehner-Hansen S, Jarrige B, Daijardin JB. Leptospirosis: prognostic factors associated with mortality. Clin Infect Dis. 1997;25:720–4. 10.1086/513767 9314467

[13] Panaphut T, Domrongkitchaiporn S, Thinkamrop B. Prognostic factors of death in leptospirosis: a prospective cohort study in Khon Kaen, Thailand. Int J Infect Dis. 2002;6:52–9. 10.1016/S1201-9712(02)90137-2 12044303

[14] Gouveia EL, Metcalfe J, de Carvalho AL, Aires TS, Villasboas-Bisneto JC, Queirroz A, Leptospirosis-associated severe pulmonary hemorrhagic syndrome, Salvador, Brazil. Emerg Infect Dis. 2008;14:505–8. 10.3201/eid1403.071064 18325275 PMC2570821

